# Investigation of Fumasep^®^ FAA3-50 Membranes in Alkaline Direct Methanol Fuel Cells

**DOI:** 10.3390/polym15061555

**Published:** 2023-03-21

**Authors:** Carmelo Lo Vecchio, Alessandra Carbone, Irene Gatto, Vincenzo Baglio

**Affiliations:** Consiglio Nazionale Delle Ricerche, Istituto di Tecnologie Avanzate per l’Energia “Nicola Giordano”, CNR-ITAE, Via Salita Santa Lucia Sopra Contesse 5, 98126 Messina, Italy

**Keywords:** anion exchange membrane, alkaline direct methanol fuel cell, Fumasep^®^ FAA-3-50, hydroxide form, methanol crossover, anion conductivity, maximum power density

## Abstract

This paper describes the use of a commercial Fumasep^®^ FAA3-50 membrane as an anion exchange membrane (AEM) in alkaline direct methanol fuel cells (ADMFCs). The membrane, supplied in bromide form, is first exchanged in chloride and successively in the hydroxide form. Anionic conductivity measurements are carried out in both a KOH aqueous solution and in a KOH/methanol mixture. AEM-DMFC tests are performed by feeding 1 M methanol, with or without 1 M KOH as a supporting electrolyte. A maximum power density of 5.2 mW cm^−2^ at 60 °C and 33.2 mW cm^−2^ at 80 °C is reached in KOH-free feeding and in the alkaline mixture, respectively. These values are in good agreement with some results in the literature obtained with similar experimental conditions but with different anion exchange membranes (AEMs). Finally, methanol crossover is investigated and corresponds to a maximum value of 1.45 × 10^−8^ mol s^−1^ cm^−2^ at 50 °C in a 1 M KOH methanol solution, thus indicating that the Fumasep^®^ FAA3-50 membrane in OH form is a good candidate for ADMFC application.

## 1. Introduction

Energy conversion from renewable sources, such as wind or sun, is the most widespread technique to obtain green electrical energy from wind turbines or solar panels. However, these systems suffer the intermittence of windy or sunny days [1,2,3]. The ever-increasing demand for global energy synergistically requires the employment of other conversion technologies, such as fuel cells (FCs), able to convert, directly and continuously, the chemical energy of a fuel into electrical energy. Versatility, high performance and facile scalability are the main characteristics of FCs that permit them to satisfy many power applications (from portable to stationary) through fuel substitution. In this context, methanol fuel can be fed to the anode of a direct methanol fuel cell (DMFC) for the electronic market application (<1.5 kW) [4,5,6,7]. The advantages of using this fuel are related to its high energy density (6100 mW h g^−1^) and easy transportation, being liquid at room temperature. With the view to establishing global decarbonization, the produced CO_2_ coming from the methanol oxidation reaction (MOR) could be recycled in a co-electrolysis cell to form methanol or chemicals again in a CO_2_-neutral cycle [8,9,10]. Another “green” alternative is to obtain methanol from biomasses or sources that need CO_2_ for their growth [11,12,13].

In an acid environment, MOR at the anode of a fuel cell is accompanied by an oxygen reduction reaction (ORR) at the cathode side, as shown in Equations (1) and (2), respectively, and described by the balanced electronic and protonic overall reaction in Equation (3).

CH_3_OH + H_2_O → CO_2_ + 6 H^+^ + 6 e^−^ (1)

3/2 O_2_ + 6 H^+^ + 6 e^−^ → 3 H_2_O (2)

CH_3_OH + 3/2 O_2_ → CO_2_ + 2 H_2_O (3)

Two redox reactions take place in face-to-face compartments, electrically separated by a polymeric membrane. Electrons, produced from alcohol oxidation at the anode, reach the cathode through an external circuit where they are gathered for the ORR. The proton exchange membrane (PEM) acts as both an electrolyte, able to exchange H^+^ between anode and cathode, and a gas separator, avoiding direct mixing of reagents, fuel (methanol) and combustive agent (oxygen). Unfortunately, a major drawback is the high methanol crossover, which lowers the performance of the cell due to the formation of a mixed potential at the cathode. One of the most commonly adopted approaches to limit methanol crossover is to introduce inorganic fillers in the polymeric matrix with the aim of increasing the tortuosity of the methanol permeation pathway and blocking the hydrophilic regions through which methanol permeates [14,15,16,17,18].

Alkaline direct methanol fuel cell (ADMFC) technology exploits the same principle of DMFC, whereas the two redox reactions, shown in Equations (4) and (5) and represented by the whole process in Equation (6), occur in an alkaline environment where the electrolyte is represented by an alkaline anion exchange membrane (AEM) able to exchange OH^−^ between electrodes [19,20,21,22]. Generally, electrochemical reactions in ADMFC are kinetically favored compared to protonic ones due to the presence of hydroxide anions at the anode that accelerates the MOR and the leakage of carbonate and bicarbonate in the outlet [23,24]. Furthermore, an alkaline environment is less corrosive than an acid medium, thus opening the possibility of employing non-precious metal catalysts for both MOR and ORR with potentially higher durability [25,26]. Methanol crossover is also reduced in AEM-DMFCs. In fact, in proton exchange membrane DMFCs, methanol can diffuse from the anode to the cathode through the membrane due to the electroosmotic drag (H^+^ ions go from the anode to the cathode, carrying water and methanol molecules), whereas in anion exchange membranes, OH^−^ ions go in the opposite direction (from the cathode to the anode), thus hindering methanol permeation.

CH_3_OH + 6 OH^−^ → CO_2_ + 5 H_2_O + 6 e^−^ (4)

3/2 O_2_ + 3 H_2_O + 6 e^−^ → 6 OH^−^ (5)

CH_3_OH + 3/2 O_2_ → CO_2_ + 2 H_2_O (6)

Contrary to conventional DMFCs, there are limited data reported in the literature about polarization and power density measurements of membrane electrode assemblies (MEAs) in an alkaline environment. Performances, achieved with different AEMs using PtRu and Pt catalysts at the anode and cathode of ADMFCs, respectively, are summarized below. Sajjad et al. [27] employed a guanidinium–chitosan (Gu-Chi2.2) blend membrane-based MEA with the achievement of an open circuit voltage (OCV) value of 0.69 V and power density of 2.0 mW cm^−2^ at room temperature with 3 M methanol/1 M KOH feed. Gupta et al. used an MEA based on KOH-doped PVA AEM [28] achieving an OCV of 0.6 V and a maximum power density of 7.1 mW cm^−2^ at 30 °C with 6 M KOH and 3 M methanol concentrations. Janarthanan et al. [24] studied different ADMFCs based on TMAC6PP AEM as a function of methanol and KOH concentrations and the best results (OCV of 0.84 V and power density of 53.8 mW cm^−2^ at 80 °C) were obtained feeding 1 M methanol–1 M KOH. Furthermore, they demonstrated that the maximum power density in a KOH-free fuel condition (3.97 mW cm^−2^) was significantly lower. Varcoe et al. achieved a peak power density of 2.8 mW cm^−2^ and an OCV of 0.48 V at 50 °C in aqueous 2 M methanol [29] with a poly(ethylene-co-tetrafluoroethylene)-based AEM. Their study also showed a higher OCV (0.7 V) and a lower performance when the thickness of the membrane was increased. Thus, it is derived that the presence of KOH in the fuel is necessary for reaching higher power densities due to an improved conductivity and electrodes kinetics favored by the high concentration of OH^−^. On the other hand, an excessive KOH concentration could damage the fuel cell hardware and it is not attractive for large-scale commercialization of this technology. One of the most relevant data, obtained with 1 M methanol–1 M KOH feed was obtained by Prakash et al. [30] by using a Tokuyama AEM-based MEA (maximum power density of 56 mW cm^−2^ at 60 °C) even if 8 mg cm^−2^ of Pt loading was employed at the anode and cathode. Santasalo et al. [31] employed an AEM from Fumatech (FAA-2) investigating its physicochemical properties and the performance in DMFCs. An OCV of 0.58 V and a maximum power density of 0.32 mW cm^−2^ were achieved at room temperature in a KOH-free anodic feed.

In our recent works, a Fumasep^®^ membrane (FAA3-50) and its ionomer (FAA3-ION) were characterized and adopted for different applications: photoelectrolysis [32], co-electrolysis [33], AEM fuel cells [34] and AEM electrolyzers [35].

In this work, the same membrane and ionomer are physicochemically characterized and investigated for direct methanol fuel cell applications. To our knowledge, for the first time, physicochemical characteristics of the Fumasep^®^ membrane (FAA3-50) are assessed for its use in the presence of methanol and KOH solutions. In particular, dimensional variation, in-plane anion conductivity, methanol uptake, methanol crossover and performance in ADMFC in the presence or absence of a supporting KOH electrolyte are performed. The results could represent the reference for future investigations on different membranes.

## 2. Materials and Methods

### 2.1. Membrane and Ionomer Preparation

A Fumasep^®^ membrane (FAA3-50 from Fumatech, Bietigheim-Bissingen, Germany) is based on a brominated polysulfone backbone with quaternary ammonium side chain groups [36,37]. It is characterized by a thickness of 50 μm and was selected for ADMFC applications.

Commercial FAA3-50, received in bromide form, was rinsed in 1 M NaCl solution for 72 h. The anionic membrane, in chloride form, was further conditioned in a fresh 1 M KOH solution for 24 h before membrane electrode assembly.

**Scheme 1:**
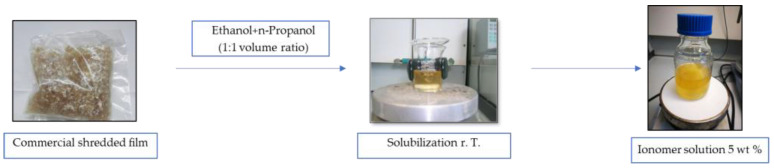
Ionomer preparation from FAA3-50 shredded membrane.

The ionomer dispersion (FAA3-ION, 5 wt.%) was obtained by solubilizing the shredded FAA3 film in a mixture of alcoholic (n-propanol and ethanol 1:1 wt.) solvents at room temperature, as described in previous papers [32,34,35] and highlighted in Scheme 1.

### 2.2. Determination of Ion Exchange Capacity (IEC), Dimensional Variation and Swelling

IEC measurements were performed by acid-base back titration as elsewhere reported [33].

λ, which is the number of hydrated hydroxide molecules per functional group, is calculated as the ratio between solution uptake and the IEC.

Dimensional variations were calculated by the difference in dimensions before and after immersion of the dried samples in aqueous or methanolic 1 M KOH solution at 30 °C for 24 h, followed by washing steps in pure water until the pH of the rinsed water is neutral. In particular, on rectangular-shaped samples, area and thickness variations were determined.

To reach the dry state, samples were maintained in an oven under vacuum (1000 mbar) for 2 h at 50 °C. The methanolic solution was prepared considering a concentration of 1 M KOH and 1 M methanol diluted in a total volume of 1 L of water. Anion concentration, expressed in M, is calculated as follows:

[OH^−^] = IEC_OH_^−^ × m_dry_/V_wet_

### 2.3. In-Plane Anion Conductivity

In-plane anion conductivity of membranes was measured by a four-electrode method by electrochemical impedance spectroscopy (EIS), as reported elsewhere [33,34]. Before measurements, membranes in chloride form were immersed in aqueous or methanolic 1 M KOH solution at 30 °C for 24 h, followed by washing steps in pure water until the pH of the rinsed water was neutral. Measurements were carried out in the range of temperature 30–80 °C, flowing fully humidified N_2_.

The activation energy was calculated by Arrhenius’s law from conductivity results. The hydroxide diffusion coefficient (cm^2^ s^−1^) was calculated as elsewhere reported [33].

### 2.4. Membrane Electrode Assembly (MEA)

For the anode, 60% PtRu/C (Alfa Aesar) was deposited onto Sigracet 39BB (SGL group) by doctor blade technique to achieve a 1.5 mg cm^−2^ Pt loading. In particular, the right amount of PtRu/C electrocatalyst was weighed and dispersed in a few milliliters of water in an ultrasonic bath. In a second step, FAA3-ION was added to the ink to obtain a 20 wt.% of ionomer with respect to PtRu/C. After 3 h in the ultrasonic bath at 50 °C, the ink was spread onto SGL 39BB and then dried overnight. Finally, PtRu/C on the carbonaceous substrate was punched to obtain a 2.3 cm × 2.3 cm electrode area.

For the cathode, 40% Pt/C (Alfa Aesar) was deposited, in a similar way, onto SGL 39BB substrate keeping a constant ionomer percentage while the amount of Pt was decreased to a 1 mg cm^−2^ loading.

### 2.5. Electrochemical Measurements

FAA3-50 anionic membrane (50 µm), in hydroxide form, was subjected to a cold assembly between anode and cathode and mounted in fuel cell hardware. Membrane electrode assembly (MEA) was surrounded by gaskets and in contact with a 5 cm^2^ flow field (graphite plates) where methanol and KOH were fed to the anode, and oxygen to the cathode side. Gold-covered plates were connected to a fuel cell station for electrochemical measurements and the cell was kept under pressure thanks to stainless steel plates blocked with a tightening force of 9 Nm.

The electrochemical apparatus consisted of a Metrohm Autolab potentiostat/galvanostat (Utrecht, The Netherlands) equipped with a Frequency Response Analyzer (FRA).

Polarization tests were carried out at different temperatures between 30 and 80 °C by feeding 1 M KOH + 1 M methanol solution to the anode side and 100 cc min^−1^ oxygen flow to the cathode side.

Electrochemical impedance spectroscopy (EIS) was carried out in the range of frequency 100 kHz–1 Hz at a cell voltage of 0.35 V.

Methanol crossover through the membrane was also investigated in the temperature range of 30–50 °C by linear sweep method. MEAs were produced using a Pt/C electrode at both the anode and cathode. 1 M KOH solution with different methanol concentrations (0.1, 0.5, 1 M) was fed to the anode side with a flow rate of 2 cc min^−1^, whereas 100 cc min^−1^ nitrogen flow was fed to the cathode side. The current at 900 mV was used for the calculation of methanol crossover.

## 3. Results and Discussion

### 3.1. Membrane Characterization

To investigate the effect of methanol on the physicochemical properties of the membrane, the uptake, λ and dimensional variation parameters were measured at 30 °C for the FAA3-50 membrane after immersion in KOH 1 M aqueous and methanol solutions, as reported in Table 1. In the first row, the values obtained in a KOH aqueous solution are shown. In the second row, data obtained after immersion of the membrane for 22 h in a 1 M KOH aqueous solution followed by immersion for 2 h in a 1 M MeOH solution (total time 24 h) are reported. In the third row, data obtained after immersion for 24 h in a methanolic 1 M KOH solution are shown. These last two data were measured to understand the behavior of the membrane in the presence of solution simulating those used during fuel cell experiments. A higher uptake was obtained when the membrane was immersed in a methanol solution. The values reported in rows 2 and 3 are the same in terms of uptake and λ, but dimensional variations significatively change when the membrane is immersed in a methanolic or aqueous solution. The presence of methanol contributes to increasing the uptake with respect to the KOH aqueous solution, with a consequent increase in the λ value, meaning that more OH^−^ molecules are coordinated to functional groups. The hydroxide concentration is the same or slightly reduced due to an effect of dilution when methanol is present since the ion exchange capacity is constant and the uptake is increased.

Regarding dimensional variations, it is evident that the excess of the solution is mainly distributed in the area rather than in the thickness. These data highlight that the methanol solution is located in different environments inside the membrane. In fact, when the ion exchange is carried out in KOH and then the membrane is immersed in methanol, the area percentage is higher, meaning that methanol is not mainly located in the conduction channels but on the entire surface of the membrane. Conversely, when the ion exchange in KOH is carried out in a methanol/KOH mixture, methanol is partly dissociated to methoxy (CH_3_O^−^), the uptake of methoxy ion diverges from that of methanol and, accordingly, these results are different [31].

**Figure 1:**
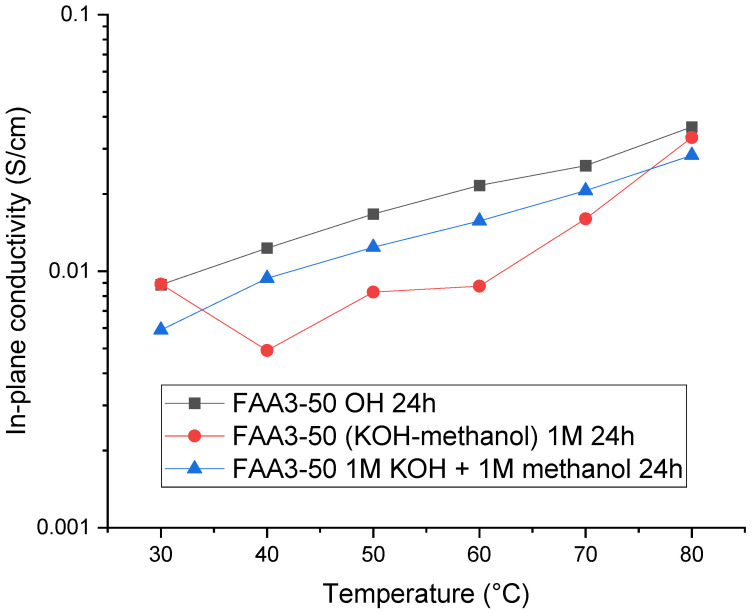
Anion conductivity of membrane after immersion in KOH or KOH/methanol or methanol after KOH for 24 h total time.

**Figure 2:**
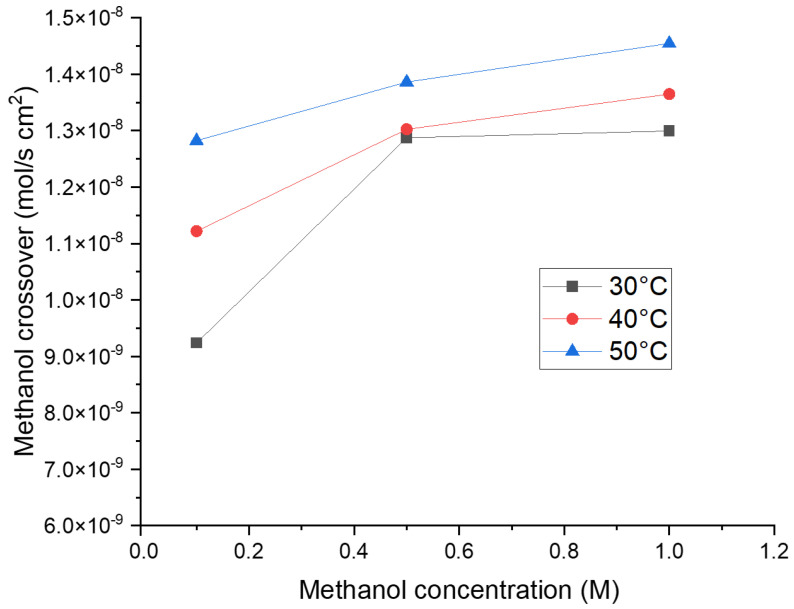
Methanol crossover values as a function of methanol concentration (in 1 M KOH solution), at different temperatures.

Figure 1 shows the in-plane conductivity of the AEM after immersion in KOH or KOH/methanol or methanol after KOH for 24 h of total time. The values shift from 5 mS cm^−1^ at low temperatures to a maximum of 36.5 mS cm^−1^ at 80 °C, in agreement with the values reported in the literature for similar or different AEMs [31,38]. The anion conductivity measured after immersion in the methanol solution is lower than in water. The membrane immersed in 1 M methanol after a 1 M KOH solution presents lower conductivity than that immersed in an aqueous KOH solution. The trend is the same, and a reduction of about 20 mS cm^−1^ was found for all the investigated temperatures. Regarding membrane exchange in a methanolic KOH solution, at the first measured temperature (30 °C), the conductivity is similar due to the high uptake of the liquid solution leading to high hydroxide solvation. Increasing the temperature, a progressive drying of excess surface water occurs and the conductivity decreases. This phenomenon could also be explained considering that the conductivity is mainly ruled by the presence of methanol and hydroxides inside the membrane. In fact, in the presence of methanol, the degree of electrolyte dissociation is lower due to methanol ion pair formation, and that results in a decreasing concentration of current carriers [39]. In addition, the probable presence of methoxy ions in the solution further reduces the ion mobility and, consequently, the conductivity. This hypothesis is also supported by the anion diffusion coefficient (D_σ_, Table 1); in fact, the values calculated at room temperature for the membrane after immersion in an aqueous solution (rows 1 and 2 of Table 1) are higher than those calculated for a methanol solution. The order of magnitude is coherently the same with the low concentration of KOH and the predominance of water in the solution. Activation energy is also in accordance with these data; in fact, similar values (27.4 kJ/mol and 26.5 kJ/mol) were calculated for membranes immersed in an aqueous solution, while it was found to be about twice (41 kJ/mol) that when the methanolic solution was used for ion exchange. In any case, the conduction mechanism is mainly ruled by the Grotthuss mechanism [33]. A further increase in temperature up to 80 °C produces a swelling of the polymer matrix and an increased ion mobility; thus, the conductivity is again comparable. Another important parameter in membranes for ADMFCs is the methanol permeability or crossover. This aspect is of particular importance in conventional DMFCs where methanol, during operation, is transported through the membrane due to the electroosmotic drag. In an alkaline environment, where OH^−^ migration is from the cathode to the anode (contrary to methanol permeation), this effect should be reduced, but it is still important to optimize performance and operating conditions, especially the methanol concentration feeding to the anode. For this reason, ex situ methanol crossover measurements were carried out at different temperatures by increasing methanol concentration from 0.1 M up to 1 M, as reported in Figure 2.

Methanol crossover increases both by increasing the temperature and concentration, even if a strong increase occurs in the range of 0.1–0.5 M instead of 0.5–1 M at a lower temperature (30 °C). In any case, the highest crossover value (1.45 × 10^−8^ mol s^−1^ cm^−2^) is in the order of magnitude reported for polyaromatic-based membranes also in an acidic environment [40]. These results are promising for the application of this membrane for ADMFC application.

### 3.2. Hardware Set-Up for AEM-DMFC Tests

**Scheme 2:**
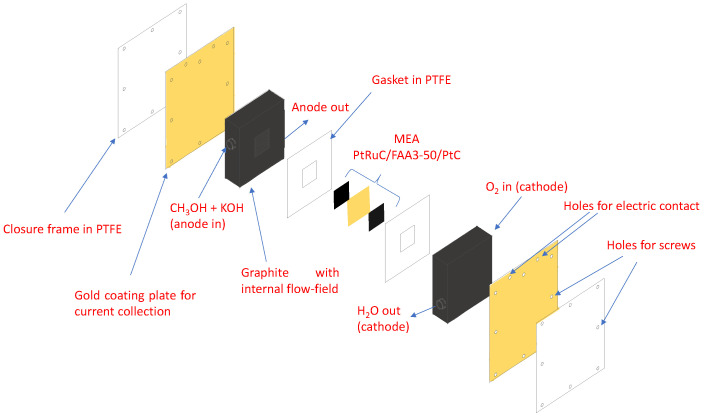
Exploded view of the hardware used for AEM-DMFC experiments.

Hardware set-up for AEM-DMFC experiments needs to be compatible with a corrosive KOH solution fed to the anode side. Thus, it was remodeled in comparison with previous hardware used in an acid environment by our group [41]. Scheme 2 shows an exploded symmetric view of 11 components employed for investigation in alkaline media. From external to internal components, the hardware includes: support plates in PTFE to allocate eight screws for tightening closure frames, and a gold coating plate for current collection through graphite plates where the anode was fed with methanol and KOH and the cathode with O_2_, directly onto the 5 cm^2^ flow field. The core is represented by membrane electrode assembly (MEA) in which PtRu/C (anode side) and Pt/C (cathode side) electrocatalysts are deposited onto gas diffusion layers. The porous backing layers of electrodes are in contact with the flow fields of the graphite plate, whereas gaskets in PTFE prevent leakage.

### 3.3. AEM-DMFC Measurements

**Figure 3:**
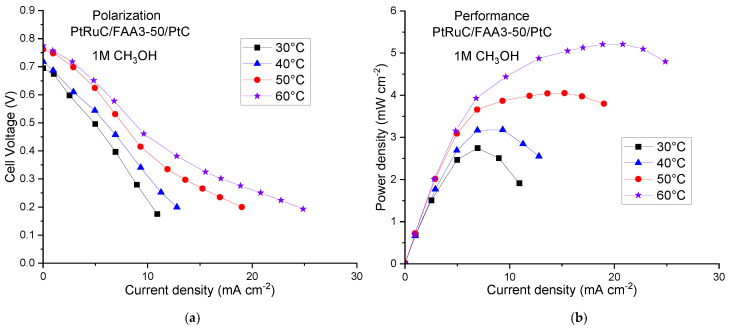
(**a**) Polarization and (**b**) power density curves of Fumasep^®^ AEM-based MEA with 1 M methanol feeding.

Figure 3 shows the polarization and power density results achieved in a range of temperatures between 30 and 60 °C, feeding a 1 M methanol solution at the anode without any addition of KOH. OCV swings between 0.7 V at 30 °C and 0.775 V at 60 °C showing a significant overpotential. Furthermore, at a high current density, the power density reaches its maximum at 60 °C with a value of 5.2 mW cm^−2^. These values are too far (at least one order of magnitude) from the results obtained in an acidic environment [42,43]. By analyzing these data, the low performance could be conferred to a KOH-free environment, as also described in the literature [24,27,28,29,30,31], considering AEMs assembled between Pt-based catalysts.

**Figure 4:**
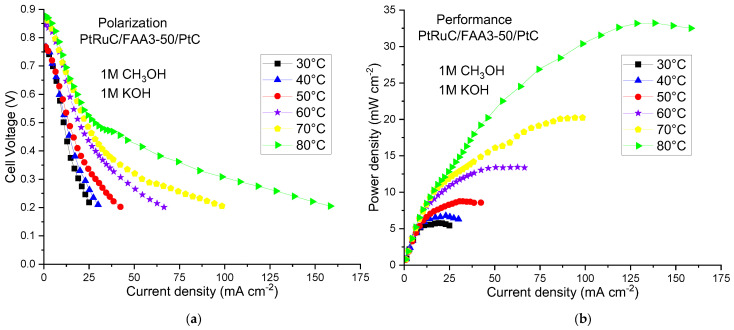
(**a**) Polarization and (**b**) power density curves of Fumasep AEM-based MEA with 1 M methanol–1 M KOH feeding.

Due to the poor performance in methanol, KOH was added to the solution to reach a 1 M concentration of a KOH/methanol mixture and to investigate the AEM-based MEA even at higher temperatures. Figure 4 shows that, at low current densities, OCV rises from 0.76–0.77 V in the range of 30–50 °C to 0.88 V at higher temperatures. The performance in ADMFC is more than double compared with KOH-free feeding between 30 and 60 °C and reaches a maximum of 33.2 mW cm^−2^ at 80 °C. The increased power density is related to both improved AEM conductivity and electrode kinetics.

**Figure 5:**
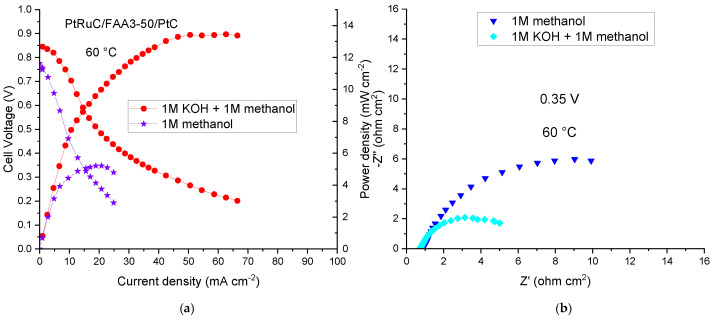
Comparison of Fumasep^®^ AEM-based MEAs at 60 °C in the presence or absence of KOH; (**a**) polarization and power density curves and (**b**) electrochemical impedance spectra.

As depicted in Figure 5a, the comparison between the MEAs, fed with or without KOH at the anode of AEM-DMFC, results in a significant difference in the three regions characterizing a polarization curve, i.e., kinetic contribution at low current density, ohmic in the intermediate values and mass transport or diffusion of reactants at high current density. Consequently, the maximum power density, achieved in the presence of KOH in the mixture (13.4 mW cm^−2^), is more than double compared to that obtained by feeding a KOH-free methanol solution (5.2 mW cm^−2^). Furthermore, electrochemical impedance spectroscopy (EIS) in Figure 5b was acquired at 0.35 V where the electrochemical reactions achieve their maximum performance. At high frequencies in the Nyquist plot, the series resistance (Rs) value is 0.72 Ω cm^2^ for ADMFC fed with a methanol/KOH mixture and 0.82 Ω cm^2^ for the MEA fed by a KOH-free methanol solution. At low frequencies, the value of charge transfer resistance (Rct) is remarkably raised for the MEA fed with a bare methanol solution due to the low presence of OH^−^, which is a reactant for the methanol oxidation reaction.

Table 2 summarizes the most important data obtained in the literature for AEM-DMFC based on Pt-electrodes and different membranes. In the literature, some comparable results were obtained by employing similar electrocatalysts but different AEMs, thus demonstrating that the FAA3-50 membrane is suitable for this application [24,27,28,30,44]. Other works reported a higher performance when non-noble metals substitute Pt-based catalysts, mainly at the cathode side [23,45,46,47] or with higher KOH concentration and catalyst loadings [38,48,49,50]. The dependence of improved performance with increasing KOH concentration was ascertained by many studies (see references in Table 2), whereas a compromise between KOH feeding and the obtainment of a less corrosive environment for hardware components is necessary. Furthermore, Galvan et al. [38] paid attention to the anion exchange ionomer (AEI) in ADMFC by investigating different species and percentages, particularly on the anode side. They demonstrated that it is possible to significantly increase the performance and reduce the Pt loading by optimizing either the interaction between the binder and catalyst nanoparticles or creating the enhanced triple-phase boundary for anion transport. In our work, a 20 wt.% AEI was adopted in the first attempt to assess the feasibility of using such a membrane and ionomer in ADMFC application. In a future study, we should analyze in detail variable AEI loadings to reach an optimized performance and stability.

## 4. Conclusions

Fumasep^®^ FAA3-50 anion exchange membranes were investigated for low and intermediate temperatures in AEM-DMFCs. The membrane exchanged in KOH showed reduced liquid uptake in an aqueous solution than in methanol with, consequently, lower swelling in terms of dimensional variations. Conversely, the conductivity of the membrane after immersion in a methanol solution (36.5 mS cm^−1^ at 80 °C) is lower than in water due to the different polarities of methanol molecules that reduced ion transport in conduction channels. In the second step, the membrane was assembled with benchmark catalysts to evaluate the performance of the AEM in DMFCs with and without KOH-supporting electrolytes at the anode. The maximum power densities of MEAs fed with 1 M methanol and 1 M KOH were 13.41 mW cm^−2^ at 60 °C and 33.2 mW cm^−2^ at 80 °C, generally, more than double compared with a KOH-free feed. Finally, crossover measurements demonstrated that methanol permeation from the anode to the cathode through the membrane corresponds to a very low value of 1.45 × 10^−8^ mol s^−1^ cm^−2^, thus indicating the feasibility of the FAA3-50 membrane being used in alkaline DMFC. Future work will consider reducing or eliminating the use of noble metals from the electrodes, lowering in this way the cost of DMFC devices. In fact, the alkaline environment should favor the kinetics of methanol oxidation and oxygen reduction reactions. However, to reach higher performances is necessary to increase the operating temperature; thus, new AEMs or hybrid/composite membranes must be developed to extend the range of applied temperatures.

## Figures and Tables

**Table 1:** Physicochemical parameters in KOH aqueous and methanolic solution.

Membrane	KOH Uptake, %	λ	A, %	Th, %	[OH], M	D_σ_, cm^2^/s	E_att_, kJ/mol
FAA3-50 (1 M KOH)	70	25	33	12	1.1	7.17 × 10^−6^	27.4
FAA3-50 1 M KOH + 1 M MeOH	86	30	56	7	1.1	1.06 × 10^−6^	26.5
FAA3-50 (1 M KOH/1 M MeOH mixture)	86	30	35	13	1.0	1.53 × 10^−6^	41.0

**Table 2:** Literature survey for AEM-DMFCs.

Reference	Working Temperature (°C)	Anode and Pt Loading (mg cm^−2^)	Anionic Exchange Membrane Electrolyte	Cathode Catalyst Loading (mg cm^−2^)	[MeOH]/ [KOH] (mol/L)	Open Circuit Voltage (V)	Maximum Power Density (mW cm^−2^)
Sajjad et al. [27]	25	Pt/C 1.0	Guanidinium–chitosan	Pt/C 1.0	3 M/1 M	0.69	2.0
Gupta et al. [28]	30	PtRu/C 1.0	KOH-doped PVA	Pt/C 1.0	3 M/6 M	0.60	7.1
Janarthanan et al. [24]	80	Pt/C 2.5	TMAC6PP	Pt/C 2.5	1 M/1 M	0.84	53.8
Janarthanan et al. [24]		Pt/C 2.5	TMAC6PP	Pt/C 2.5	1 M/KOH-free	0.56	3.97
Varcoe et al. [29]	50		Poly(ethylene-co-tetrafluoro ethylene)		2 M/KOH-free	0.48	2.16
Prakash et al. [30]	60	PtRu 8.0	Tokuyama	Pt 8.0	1 M/1 M	0.79	56.0
Zhang et al. [44]	65	PtRu/C 1.0	AQPVBH	Pt/C 1.0	1 M/1 M NaOH	0.71	53.2
Galvan et al. [38]	60	PtRu 0.7	TPN	Pt 1.3	2 M/4 M	0.71	151
Galvan et al. [38]	60	PtRu 0.7	TPN	Pt 1.3	2 M/KOH-free	0.58	20
Santasalo et al. [31]	30	PtRu/C 1.0	FAA-2 Fumatech	Pt/C 1.0	1 M/KOH-free	0.58	0.32
This work	60	PtRu/C 1.5	FAA3-50 Fumatech	Pt/C 1.0	1 M/1 M	0.845	13.4
This work	70	PtRu/C 1.5	FAA3-50 Fumatech	Pt/C 1.0	1 M/1 M	0.868	20.2
This work	80	PtRu/C 1.5	FAA3-50 Fumatech	Pt/C 1.0	1 M/1 M	0.874	33.2
This work	60	PtRu/C 1.5	FAA3-50 Fumatech	Pt/C 1.0	1 M/KOH-free	0.775	5.2

## Data Availability

The data presented in this study are available on request from the corresponding author.
